# rTMS over the human medial parietal cortex impairs online reaching corrections

**DOI:** 10.1007/s00429-023-02735-7

**Published:** 2023-12-23

**Authors:** Rossella Breveglieri, Sara Borgomaneri, Annalisa Bosco, Matteo Filippini, Marina De Vitis, Alessia Tessari, Alessio Avenanti, Claudio Galletti, Patrizia Fattori

**Affiliations:** 1 Department of Biomedical and Neuromotor Sciences, University of Bologna, Piazza di Porta S. Donato 2, 40126 Bologna, Italy; 2 Center for studies and research in Cognitive Neuroscience, Department of Psychology, University of Bologna, Cesena Campus, 47521 Cesena, Italy; 3 Alma Mater Research Institute For Human-Centered Artificial Intelligence (Alma Human AI), University of Bologna, Bologna, Italy; 4 Department of Psychology, University of Bologna, 40127 Bologna, Italy; 5 Center for research in Neuropsychology and Cognitive Neurosciences, Catholic University of Maule, 3460000 Talca, Chile

**Keywords:** Correction of reach direction and position, Posterior parietal cortex, Human brain, Area V6A, Limb state estimation

## Abstract

Indirect correlational evidence suggests that the posteromedial sector of the human parietal cortex (area hV6A) is involved in reaching corrections. We interfered with hV6A functions using repetitive transcranial magnetic stimulation (rTMS) while healthy participants performed reaching movements and in-flight adjustments of the hand trajectory in presence of unexpected target shifts. rTMS over hV6A specifically altered action reprogramming, causing deviations of the shifted trajectories, particularly along the vertical dimension (i.e., distance). This study provides evidence of the functional relevance of hV6A in action reprogramming while a sudden event requires a change in performance and shows that hV6A also plays a role in state estimation during reaching. These findings are in line with neurological data showing impairments in actions performed along the distance dimension when lesions occur in the dorsal posterior parietal cortex.

**Supplementary Information:**

The online version contains supplementary material available at 10.1007/s00429-023-02735-7.

## Introduction

Interacting with objects in different spatial positions largely relies on the ability to correct in-flight reaching movements. There is a large consensus regarding the functional role of the posterior parietal cortex (PPC) in the adjustment of motor commands based on the estimation of limb state during reaching (Wolpert et al. 1998; Mulliken et al. 2008; Vallar and Coslett 2018; Medendorp and Heed 2019). The first supporting evidence came from patients with PPC lesions, showing inaccurate reaching toward peripheral visual targets (optic ataxia) (Perenin and Vighetto 1988; Rossetti et al. 2019). This inability suggests that the PPC is responsible for correction of the arm movement (‘automatic pilot’ (Pisella et al. 2000; Gréa et al. 2002)), but the lesion responsible for these impairments was typically very large, ‘involving Brodmann’s areas 18, 19, 7, 39, as well as the intraparietal sulcus of both hemispheres’ (Pisella et al. 2000), making it difficult to interpret the specific role of the different subregions of the PPC in the control of reaching.

Transcranial magnetic stimulation (TMS) (Pitcher et al. 2021) can help to establish a causal role for specific subregions of the PPC. In a seminal study by Desmurget and colleagues (Desmurget et al. 1999), single-pulse TMS was delivered over the lateral part of the superior parietal lobule in the PPC of healthy participants during a look-and-point reaching movement directed at a target that could stay still or unexpectedly jump to a new location. In line with the abovementioned lesion studies (Pisella et al. 2000; Gréa et al. 2002), this study elegantly demonstrated that TMS over PPC affected the ability of participants to correct the direction of arm movement, which resulted in a lower reaching precision, thus confirming the causal role of PPC in online reaching corrections (Desmurget et al. 1999). These results, however, are still under debate. First of all, Desmurget himself showed that one of his participants, who had been stimulated more medially than the others, showed a slightly different pattern of results (Desmurget et al. 1999). In addition, subsequent studies did not observe any suppression of movement adjustments even if they stimulated the same area as in Desmurget’s study (Johnson and Haggard 2005; Savoie et al. 2020). Lastly, another TMS study targeting more medial/anterior parts of the PPC did not show any effect of the stimulation (Marigold et al. 2019). It is worth noting, however, that the medial posterior part of the PPC was never stimulated in these studies.

Data in favor of the role of PPC in the online correction of arm reaching also come from single cell recording studies in monkeys. Archambault and colleagues (Archambault et al. 2009) found PPC cells whose pattern of activity was strictly related to the change in trajectory that occurred when monkeys updated their reaching after a jump of target location. A reversible inactivation experiment (muscimol injection) performed in the same region confirmed these results (Battaglia-Mayer et al. 2013). Even in these studies, the investigated part of the brain was the lateral and the anteromedial part of PPC, omitting the medial posterior part of it, even if it is known that this latter contains reach-related neurons modulated before and during reaching toward stationary targets (Hadjidimitrakis et al. 2014, 2022; Bosco et al. 2016). No study to date has investigated the causal role of the posteromedial part of the PPC (that includes area hV6A; (Tosoni et al. 2015; Gamberini et al. 2020)) in the online control of reaching corrections.

To fill this gap of knowledge and to find the causal role of hV6A in reaching corrections, here we aimed to investigate the functional relevance of the medial posterior part of PPC during reaching corrections after unexpected target jumps. We also investigated the effect of stimulation on corrective movements along the vertical direction, the distance, an experiment never performed, to our knowledge, in TMS studies. To test the anatomical specificity of hV6A perturbation in altering reaching corrections, we also targeted a visual area not involved in visuomotor processes (i.e., the primary/secondary visual cortices, V1/V2), a control area widely used in TMS studies (Kamitani and Shimojo 1999; Urgesi et al. 2004; Serino et al. 2011; Kaderali et al. 2015; Breveglieri et al. 2021). The choice of this region as an active control area was motivated by the visual nature of the task, in which reaching corrections were elicited by visual shifts of the target: while V1/V2 performs basic processing of visual information, hV6A is supposed to associate visual input with motor-related ones to monitor the arm state during movement. We thus expected to find different patterns of results after active stimulations of these areas.

## Materials and methods

### Participants

Twenty-eight healthy volunteers (16 in Experiment 1: 8 males; age range 20–29 years; mean age 24.0 ± 2.3 years; 12 in Experiment 2: 3 males; age range 20–26 years; mean age 22.7 ± 2.5 years) participated in this study.

Participants were right-handed according to a standard handedness inventory (Briggs and Nebes 1975), had normal or corrected-to-normal visual acuity in both eyes, and were naïve as to the purposes of the experiment. None of the participants had neurological, psychiatric, or other medical problems, nor did they have any contraindications to TMS (Rossi et al. 2009). Participants provided written informed consent. The procedures were approved by the Bioethical Committee at the University of Bologna (Prot. 170133, Prot. 237243, Prot. 57635) and were in accordance with the ethical standards of the 2013 Declaration of Helsinki. No discomfort or adverse effects during TMS were reported or noticed.

### Localization of brain sites

The coil position was identified on each participant’s scalp using the SofTaxic Navigator system in Experiment 1 (EMS, Bologna, Italy) (Carducci and Brusco 2012; Avenanti et al. 2018; Paracampo et al. 2018), and the Cortexplore Neuronavigator (Cortexplore, Linz, Austria) in Experiment 2 (Klink et al. 2021).

In Experiment 1, we tested 2 active stimulation sites, the area of interest (left hV6A), a control area (V1/V2), and one Sham condition. In Experiment 2, we performed hV6A and Sham stimulations. In both experiments, the Talairach coordinates for hV6A we used were *x* =  − 10, *y* = − 78, *z* = 40 (Talairach and Tournoux 1988; Ciavarro et al. 2013; Breveglieri et al. 2021), that were similar to those used for studying the anterior part of the superior parieto-occipital cortex (Vesia et al. 2010, 2017), a region that likely includes hV6A (Pitzalis et al. 2015) and was investigated in several imaging studies (Filimon et al. 2009; Cavina-Pratesi et al. 2010; Gallivan et al. 2011; Tosoni et al. 2015). To target V1/V2 in Experiment 1, the coil was centered 2 cm above the center of the inion, thus resulting in a bilateral stimulation (Romei et al. 2016; Chiappini et al. 2018). In both experiments, Sham stimulation was performed by placing the coil tilted at 90° over the vertex, so that participants could feel coil–scalp contact and discharge noise as during active stimulation, but no current was induced in the brain (Lisanby et al. 2001; Sandrini et al. 2011).

### TMS protocol

Biphasic TMS pulses (10 Hz, 3 pulses, as performed in other studies on the medial PPC; (Vesia et al. 2010; Striemer et al. 2011)) were delivered using a MagStim Rapid2 stimulator (Experiment 1) or a Deymed DuoMAG XT stimulator (Experiment 2) connected to a 70 mm figure-of-eight coil. Stimulation of hV6A was carried out by placing the coil tangentially over the marked scalp sites along a parasagittal line with the handle pointing downward (Vesia et al. 2010; Breveglieri et al. 2021).

In Experiment 1, the control area (V1/V2) was targeted by placing the coil tangentially over the marked scalp sites along a parasagittal line with the handle pointing downward.

In both experiments and for all the stimulation conditions, the intensity of magnetic stimulation was fixed at 60% of the maximal stimulator output, as in several previous TMS studies targeting the PPC (Lewald et al. 2002; Dambeck et al. 2006; Vesia et al. 2006, 2010; Prime et al. 2008; Buelte et al. 2008; Delle Monache et al. 2017) and the occipital cortex (Silvanto et al. 2005; Laycock et al. 2007; Pitcher et al. 2009; Mullin and Steeves 2011; Ganaden et al. 2013).

### Apparatus and behavioral task

**Fig. 1:**
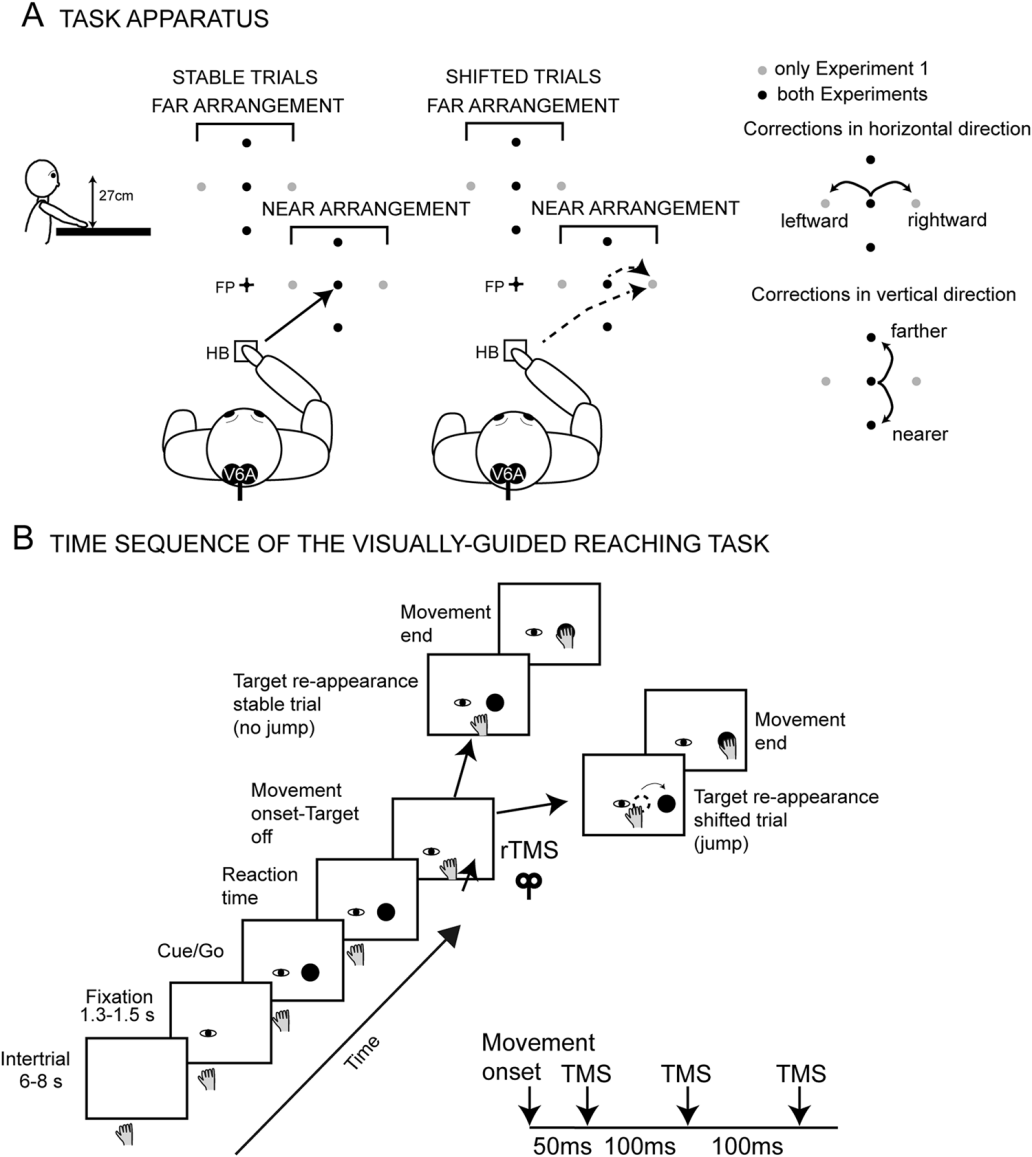
Experimental setup. **A** Lateral (left) and top (right) view of the target arrangements in the experimental task. The participants performed reaching movements with their right hand toward one of the targets (black dots represent the targets of both Experiments, whereas the gray dots correspond with the targets used only in Experiment 1) located at different distances and directions in two target arrangements: FAR arrangement (top) and NEAR arrangement (right) while fixating a fixation point (FP, cross). Reaching movements were performed from the initial hand position (Home button, HB). In the stable target trials, reaching was directed to one of the ten stationary targets (left). In the shifted target trials (right), reaching was directed to the central target in each arrangement but was suddenly reprogrammed and redirected toward a new location of the target within the same arrangement. The new target location could be in a different horizontal direction (shift in horizontal direction, only in Experiment 1) or at a different vertical direction (shift in vertical direction, in both Experiments) within each arrangement of targets. **B** Time sequence of the task in both Experiments (only one target position is shown for conciseness). The eye represents the fixation point; the filled black circle shows the reaching target. The fixation point stayed visible for 1.3 or 1.5 s and then the reaching target was turned on in one of the locations. Immediately, the participant reached for the target with her/his right hand while maintaining his/her gaze on the fixation point. Movement onset triggered the target switching off. The target appeared again at the previous location (stable target trials) or in another location (shifted target trials), requiring the participants to correct the movement online. During the movement time, rTMS was delivered with a time-course sketched below the timeline

We tested the influence of TMS of the hV6A on online reaching corrections using an apparatus (Bosco et al. 2017; Breveglieri et al. 2021) which consisted of a 19-inch touchscreen (ELO IntelliTouch 1939L, frame rate of 60 Hz) laid horizontally at waist level. In all trials, participants started the reaching movement with their right hand on a button (home-button, HB in Fig. 1A). The stimuli were green (fixation point, diameter 0.3 cm) and red (reaching target, diameter 1.2 cm) dots, the latter presented at different distances (Experiments 1 and 2) and directions (only Experiment 1) (Fig. 1A).

We sought to investigate reaching near the participants and in the ipsilateral hemispace with respect to the hand used (NEAR arrangement of targets, Fig. 1A). Another sector was located farther from the participants and along the midline (FAR arrangement of targets, Fig. 1A). Monkey studies (Hadjidimitrakis et al. 2014, 2022; Fattori et al. 2017) and a prior TMS experiment (Breveglieri et al. 2021) demonstrated that the distance from the body of a reaching target is the most effective factor that modulates V6A activity. To evaluate the differential influence of targets located at different distances or directions from the body during reaching corrections, in the current experiments we wanted to test movement deviations performed in the vertical direction or in the horizontal direction in different spatial sectors defined by the FAR and NEAR arrangements of targets (Fig. 1). In agreement with the above-mentioned studies (Hadjidimitrakis et al. 2014, 2022; Fattori et al. 2017; Breveglieri et al. 2021), we expected to find stronger effects of hV6A stimulation for corrections in the vertical direction, i.e. when correcting movements were performed to reach to targets at different distances from the body.

In each arrangement, the targets could appear in 5 locations arranged in a square with a central target and another 4 targets located 7 cm apart from each other (Experiment 1), or 3 possible locations arranged in a vertical line with a central target and another 2 targets located 6 cm apart from each other (Experiment 2); the fixation point (FP in Fig. 1A) was located at 36 cm (Experiment 1) or 33 cm (Experiment 2) away from the participant’s chest along the midline (Fig. 1A).

The sequence of visually guided reaching was the same for the 2 target arrangements (Fig. 1B) and for the two Experiments and consisted of an intertrial period (6, 7, or 8 s, randomized), followed by the presentation of the FP that prompted the participant to press the HB. Then, the participant had to stare at the FP for a randomly chosen period (1.3 or 1.5 s). After this period, the reaching target appeared, and this indicated: (i) the position to reach toward; (ii) that the participant had to promptly reach that target position while maintaining fixation on the FP. The subsequent movement onset triggered the disappearance of the target that reappeared after 20 ms, and the TMS. The target could reappear in the same location (stable target trials) or in a different location (shifted target trials).

We chose to constrain the participant’s fixation on a stable FP dissociated from the position of the reaching target during reaching execution because in a previous study (Breveglieri et al. 2021) we demonstrated that the stimulation of hV6A during reach planning was effective only if the fixation was constrained on a point dissociated from the position of the reaching target. Moreover, we wanted to avoid participants’ saccades during the movement, to rule out any possible confounds in the data interpretation given that in macaque the saccades and any change in eye position modulated the firing rate of V6A neurons (Galletti et al. 1995; Kutz et al. 2003; Breveglieri et al. 2012).

In Experiment 1, we presented < 25% (21%) of shifted target trials to make the shift of the reaching target unexpected for the participant (Posner 1980; Paulignan et al. 1991; Rumiati and Humphreys 1998; Pisella et al. 2000; Tessari and Rumiati 2004; Jacquet et al. 2012; Song et al. 2014). In Experiment 2, we lowered the number of conditions (10 reaching conditions for each of the 2 stimulation conditions) to reach a higher number of shifted target trials.

In Experiment 1, the task was composed of 15 blocks of 38 trials each (30 stable target and 8 shifted target each) for a total of 570 trials performed over the same experimental session. In Experiment 2, the task was composed of 8 blocks of 60 trials each (48 stable target and 12 shifted target each) for a total of 480 trials. We randomized the target positions, the trial types (shifted target, stable target) in each block and the blocks of each stimulation site. For stimuli presentation and data analysis, we used Matlab (Mathworks, USA, RRID: SCR_001622) with the Psychophysics toolbox extension (Brainard 1997).

### Data acquisition and analysis

The kinematics of reaching movements was recorded by sampling the position of two markers at a frequency of 100 Hz using a motion tracking system (VICON motion capture system, Vero 2.2 cameras, 2.2MP, 2048 × 1088 pixel resolution); markers were attached to the wrist (on the scaphoid bone) and the nail of the right index finger (reaching finger). Given the different duration of trials, we normalized each trajectory length by expressing each time point in % of movement time of that trial. Reaching onset was detected by the release of the HB. Reaching end time was detected by the touch on the touchscreen. Movement time was obtained by subtracting the movement onset from the respective movement end time.

To determine whether TMS affected reaching trajectories, we calculated the euclidean distance (ED) of each trajectory point between the trajectory of movement directed toward each of the peripheral target positions in each arrangement of targets and the trajectory of movements directed toward the central position of that arrangement. ED was proposed as a distance measure between time series, and since trajectories are closely related to time series, the ED was adopted in measuring trajectory distance (Su et al. 2020). For each couple of normalized trajectories and in each data point, the ED was calculated as follows:

$${\text{ED}}_{{\text{i}}} {\mkern 1mu} = {\mkern 1mu} {\text{sqrt((xmeanT}}_{{\text{i}}} - {\text{xmeanTC}}_{{\text{i}}} )^{{ \wedge 2}} {\mkern 1mu} + {\mkern 1mu} {\text{(ymeanT}}_{{\text{i}}} - {\text{ymeanTC}}_{{\text{i}}} )^{{ \wedge 2}} {\mkern 1mu} + {\mkern 1mu} {\text{(zmeanT}}_{{\text{i}}} - {\text{zmeanTC}}_{{\text{i}}} )^{{ \wedge 2}} )$$

where xmeanT(i) is the mean value (across trials) of the horizontal component of the ith data point of the lateral trajectory (the trajectory of the movement toward one of the peripheral target positions in each arrangement) and xmeanTC(i) is the mean value of the horizontal component of the ith data point of the central trajectory of the same arrangement; ymeanT(i) is the mean value of the sagittal component of the ith data point of the lateral trajectory and ymeanTC(i) is the mean value of the sagittal component of the ith data point of the central trajectory of the same arrangement; zmeanT(i) is the mean value of the elevation component of the ith data point of the lateral trajectory and zmeanTC(i) is the mean value of the elevation component of the ith data point of the central trajectory of the same arrangement.

To investigate the time-course of the effect of the stimulation on hand displacements during movements, we divided the hand displacement data points (measured as ED) into 10% time bins relative to the movement duration. Skewness and kurtosis of ED data were within the acceptable range to prove normal distribution (George and Mallery 2010), so a repeated measures analysis of variance (ANOVA) with Newman–Keuls post hoc comparisons was performed to evaluate statistical comparisons. The factor used in the ANOVA were Stimulation site (Experiment 1: SHAM, V1/V2, hV6A; Experiment 2: SHAM, hV6A), Trial type (shifted target, stable target), Position (Experiment 1: farther, nearer, rightward, leftward; Experiment 2: farther, nearer), Time bin (bin1 to bin10). Statistical analyses were performed with STATISTICA (version 10, Statsoft).

## Results

We designed Experiment 1 to investigate the causal role of hV6A in movement reprogramming after unexpected target shifts in the distance and direction dimensions (Fig. 1A).

To test functional specificity, we compared the reaching performance following rTMS over hV6A with two control rTMS conditions: SHAM and V1/V2 stimulation. In all stimulation conditions, reaching movements systematically covered a large spatial sector, resulting in 18 reaching conditions (Fig. 1A). To test the robustness of the findings from Experiment 1, in Experiment 2 we restricted our focus to a set of critical conditions, using a larger number of trials per condition. We compared the effect of rTMS over hV6A with that of SHAM rTMS—serving as a control stimulation—and focused on movements covering a smaller spatial sector (thus reducing the reaching conditions to 10) to increase the number of shifted target trials per condition.

In both Experiments, movement time was not affected by the stimulation, either in the FAR arrangement (Exp.1: all *F* < 0.64, all *p* > 0.69, all partial *η*^2^ < 0.04, Exp2: all *F* < 3.61, all *p* > 0.08, all partial *η*^2^ < 0.25, Tables 1, 2) or in the NEAR arrangement (Exp1, all *F* < 1.31, all *p* > 0.25, all partial *η*^2^ < 0.08; Exp2, all *F* < 1.92, all *p* > 0.19, all partial *η*^2^ < 0.14, Tables 1, 2), so the stimulation did not alter the motor strategy used by participants in the different stimulation conditions.

**Table 1:** Movement times in the conditions of Experiment 1

Targets	Stimulation condition	Type of trial	Position	Mean MT	SE
FAR	SHAM	SHIFTED TARGET	NEAR	897.83	52.29
FAR	SHAM	SHIFTED TARGET	RIGHT	1006.98	44.45
FAR	SHAM	SHIFTED TARGET	LEFT	892.85	29.23
FAR	SHAM	SHIFTED TARGET	UP	1011.80	50.50
FAR	SHAM	STABLE TARGET	NEAR	846.22	36.30
FAR	SHAM	STABLE TARGET	RIGHT	986.15	49.33
FAR	SHAM	STABLE TARGET	LEFT	872.03	44.79
FAR	SHAM	STABLE TARGET	UP	915.34	40.07
FAR	V1/V2	SHIFTED TARGET	NEAR	888.86	49.26
FAR	V1/V2	SHIFTED TARGET	RIGHT	1003.99	47.53
FAR	V1/V2	SHIFTED TARGET	LEFT	912.89	37.26
FAR	V1/V2	SHIFTED TARGET	UP	984.63	28.56
FAR	V1/V2	STABLE TARGET	NEAR	847.52	39.85
FAR	V1/V2	STABLE TARGET	RIGHT	989.11	42.55
FAR	V1/V2	STABLE TARGET	LEFT	856.75	37.88
FAR	V1/V2	STABLE TARGET	UP	910.12	36.09
FAR	hV6A	SHIFTED TARGET	NEAR	877.10	34.27
FAR	hV6A	SHIFTED TARGET	RIGHT	1002.39	39.16
FAR	hV6A	SHIFTED TARGET	LEFT	904.28	42.97
FAR	hV6A	SHIFTED TARGET	UP	993.39	38.59
FAR	hV6A	STABLE TARGET	NEAR	854.83	34.94
FAR	hV6A	STABLE TARGET	RIGHT	945.80	33.73
FAR	hV6A	STABLE TARGET	LEFT	863.20	38.18
FAR	hV6A	STABLE TARGET	UP	916.94	28.34
NEAR	SHAM	SHIFTED TARGET	NEAR	828.96	39.57
NEAR	SHAM	SHIFTED TARGET	RIGHT	895.26	37.84
NEAR	SHAM	SHIFTED TARGET	LEFT	915.14	47.78
NEAR	SHAM	SHIFTED TARGET	UP	884.56	44.15
NEAR	SHAM	STABLE TARGET	NEAR	815.90	40.93
NEAR	SHAM	STABLE TARGET	RIGHT	815.16	37.01
NEAR	SHAM	STABLE TARGET	LEFT	815.83	38.47
NEAR	SHAM	STABLE TARGET	UP	774.58	37.77
NEAR	V1/V2	SHIFTED TARGET	NEAR	843.65	39.68
NEAR	V1/V2	SHIFTED TARGET	RIGHT	916.13	38.62
NEAR	V1/V2	SHIFTED TARGET	LEFT	900.66	44.26
NEAR	V1/V2	SHIFTED TARGET	UP	907.04	38.45
NEAR	V1/V2	STABLE TARGET	NEAR	794.85	31.07
NEAR	V1/V2	STABLE TARGET	RIGHT	812.77	38.55
NEAR	V1/V2	STABLE TARGET	LEFT	808.95	39.23
NEAR	V1/V2	STABLE TARGET	UP	761.85	33.23
NEAR	hV6A	SHIFTED TARGET	NEAR	828.60	37.37
NEAR	hV6A	SHIFTED TARGET	RIGHT	945.89	53.89
NEAR	hV6A	SHIFTED TARGET	LEFT	876.15	39.82
NEAR	hV6A	SHIFTED TARGET	UP	873.14	36.89
NEAR	hV6A	STABLE TARGET	NEAR	780.08	37.80
NEAR	hV6A	STABLE TARGET	RIGHT	815.66	42.97
NEAR	hV6A	STABLE TARGET	LEFT	807.77	36.67
NEAR	hV6A	STABLE TARGET	UP	760.55	32.49

*MT* movement time (ms), *SE* standard error

**Table 2:** Movement times in the conditions of Experiment 2

Targets	Stimulation condition	Type of trial	Position	Mean MT	SE
FAR	SHAM	SHIFTED TARGET	NEAR	595.92	32.25
FAR	SHAM	SHIFTED TARGET	FAR	703.70	35.95
FAR	SHAM	STABLE TARGET	NEAR	590.91	31.58
FAR	SHAM	STABLE TARGET	FAR	681.00	40.55
FAR	hV6A	SHIFTED TARGET	NEAR	599.55	30.95
FAR	hV6A	SHIFTED TARGET	FAR	707.96	32.45
FAR	hV6A	STABLE TARGET	NEAR	590.70	30.71
FAR	hV6A	STABLE TARGET	FAR	669.27	36.32
NEAR	SHAM	SHIFTED TARGET	NEAR	540.34	32.60
NEAR	SHAM	SHIFTED TARGET	FAR	615.36	28.68
NEAR	SHAM	STABLE TARGET	NEAR	505.07	29.15
NEAR	SHAM	STABLE TARGET	FAR	570.83	32.05
NEAR	hV6A	SHIFTED TARGET	NEAR	536.98	29.14
NEAR	hV6A	SHIFTED TARGET	FAR	625.60	25.80
NEAR	hV6A	STABLE TARGET	NEAR	507.29	30.93
NEAR	hV6A	STABLE TARGET	FAR	569.04	29.60

Conventions as in Table 1

### Experiment 1

**Fig. 2:**
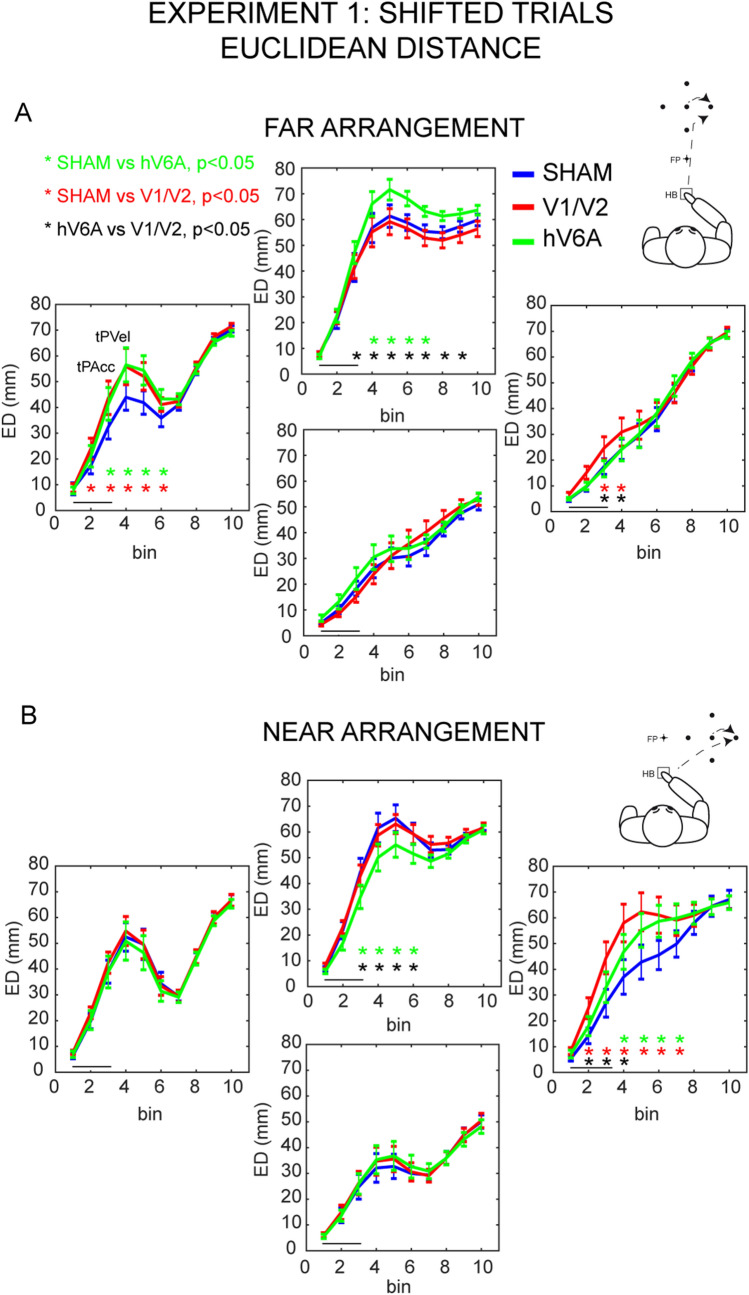
Impairments in the amount of trajectory deviations in shifted target trials of Experiment 1. Mean population euclidean distance (ED) between the shifted trajectory and the corresponding central route of the FAR arrangement of targets (**A**) and of the NEAR arrangement of targets (**B**). Within each arrangement, data are separated for direction of shift, stimulation sites, and time bins. The black line below the plot in each graph represents the stimulation time. Top left inset: tPAcc = time of maximum acceleration and tPVel = time of maximum velocity (shown only once, because the time bins of their occurrence were the same in all positions). Asterisks indicate significant post hoc comparisons (green: comparison hV6A vs SHAM; red: comparison V1/V2 vs SHAM; black: comparison hV6A vs V1/V2). In each plot, a larger area under the curve should be interpreted as a larger deviation from the central trajectory. To the right of each inset a schematic representation of the task is shown. Other conventions as in Fig. 1

The reaching trajectories (measured as ED) in the shifted target conditions were consistently influenced by TMS. Stimulations of hV6A and V1/V2 produced distinct trajectory alterations across the two arrangements of targets. In the FAR arrangement, the ED of trajectories was significantly modulated by the interaction Stimulation site x Trial type x Position x Time bin (F_54,810_ = 1.57, *p* = 0.006, partial *η*^2^ = 0.09, Fig. 2A, individual participants’ data in Fig. S1A).

This effect was driven by differential influences of the stimulation of V1/V2 and hV6A that was specific to shifted trajectories, as no reliable effect of brain stimulation was observed on stable target trials (Fig. S2). The stimulation of V1/V2 produced significant alterations of the shifted trajectories only when the jump shifted the target leftward or rightward (i.e., online correction in the horizontal direction). As shown in Fig. 2A, in rightward corrections, stimulation of V1/V2 induced a small but significant increase of ED compared to SHAM stimulation in the first part of the movement (from 20 to 40% of the movement time, all *p* < 0.04), whereas no effects were evident after hV6A stimulation (all bins *p* > 0.88). In leftward corrections, we observed a significant ED increase following the stimulation of V1/V2 and of hV6A. The stimulation of V1/V2 caused a deviation from SHAM (Fig. 2A) (V1/V2 vs. SHAM, from 10 to 60% of the movement time, all bins p < 0.04) that was earlier than that observed after hV6A stimulation (from 20 to 60% of the movement time, all bins *p* < 0.02). The effects after V1/V2 and hV6A stimulations during leftward corrections were comparable (Fig. 2A; all *p* > 0.73). Interestingly, shifted trajectories in the vertical direction were affected only by the stimulation of hV6A (Fig. 2A). In particular, the changes of ED were significant during farther corrections. The significant differences were in the central phase of the movement after the time of maximum velocity (tPVel, top left inset in Fig. 2A), which occurred after the time of peak of acceleration (tPAcc, top left inset in Fig. 2A), a well-known landmark of the end of the first ballistic phase of movement (Pélisson et al. 1986) (hV6A vs. SHAM, from 30 to 70% of the movement time, all *p* < 0.02, hV6A vs. V1/V2, from 20 to 90% of the movement time, all *p* < 0.02, Fig. 2A). In nearer corrections, which required the extent of the trajectory to be reduced to correct the movement, changes in the ED were observed but they did not reach the threshold for significance (all *p* > 0.05 across bins).

In the NEAR arrangement, we observed a pattern of effects that was similar to the one shown in the FAR arrangement, with effects during corrections in the horizontal direction for the stimulation of V1/V2, and effects during corrections along the vertical and in the horizontal directions for hV6A stimulation (F_54,810_ = 1.71, *p* = 0.001, partial *η*^2^ = 0.10, Fig. 2B and S1B). During rightward corrections, stimulation of V1/V2 caused higher deviations of the shifted trajectory in comparison to both SHAM (from 10 to 60% of the movement time, *p* < 0.002) and hV6A (from 10 to 30% of the movement time, *p* < 0.04), which in turn also differed from one another (from 30 to 60% of the movement time, all *p* < 0.003). Similar to what was observed in the FAR arrangement, in the NEAR arrangement the stimulation of hV6A also caused a significant change in trajectory during farther corrections compared to both SHAM (from 20 to 60% of the movement time, all *p* < 0.02) and V1/V2 stimulation (from 20 to 60% of the movement time, all *p* < 0.04), that in turn were not significantly different (all *p* > 0.93), and smaller, non-significant trajectory deviations in nearer corrections.

### Experiment 2

**Fig. 3:**
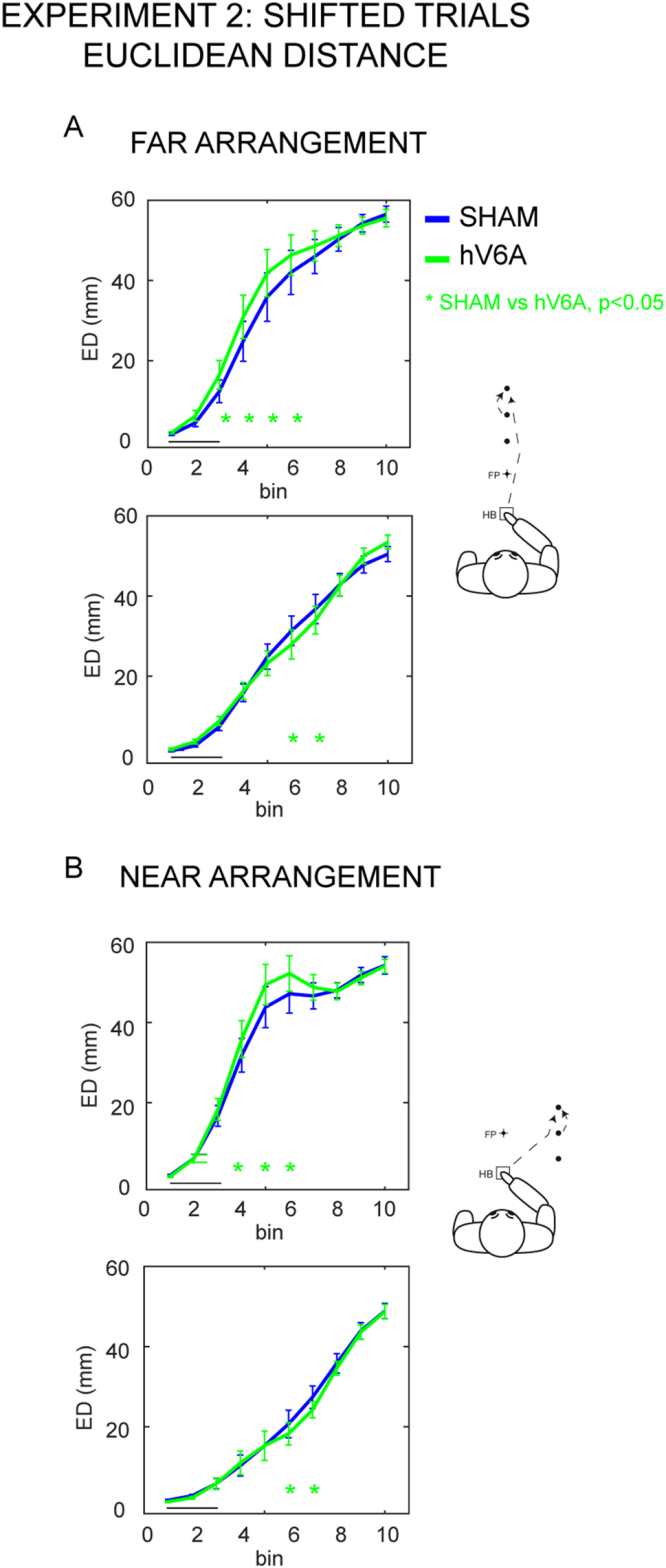
Impairments in the amount of trajectory deviations in shifted target trials of Experiment 2. Conventions as in Fig. 2

In line with Experiment 1, in Experiment 2 stimulations of hV6A produced trajectory alterations in both arrangements of targets. The ED of the trajectories of the FAR arrangement was significantly modulated by the four-way interaction (F_9,99_ = 4.53, *p* < 0.001, partial *η*^2^ = 0.29, Fig. 3A, Fig. S3). This effect was driven by differential influences of the hV6A stimulation that was specific to shifted trajectories. No reliable effect of brain stimulation was observed on stable target trials (Fig. S4), again in line with the results of Experiment 1.

The ED of the shifted trajectories was consistently influenced by rTMS over hV6A. In farther corrections, the significant differences were in the central phases of the movement (hV6A vs. SHAM, from 20 to 60% of the movement time, all *p* < 0.001, Fig. 3A, all the other p > 0.10). The small changes in the ED of the nearer corrections observed in Experiment 1 became significant in Experiment 2 (from 50 to 70% of the movement time, all *p* < 0.02; all the other *p* > 0.18) and were delayed compared to the changes for farther corrections. No effects of the stimulation were found in stable target trials (all *p* > 0.48, Fig. S4).

In the NEAR arrangement of targets, we observed the same pattern of effects that were specifically observed during shifted target trials (F_9,99_ = 3.43, *p* = 0.001, partial *η*^2^ = 0.23, Fig. 3B and S3). Similar to what was observed in the FAR arrangement, the stimulation of hV6A caused a change in the trajectory during farther corrections also in the NEAR arrangement (from 30 to 60% of the movement time, all *p* < 0.001, all others *p* > 0.20, Fig. 3B) and during nearer corrections, again with a delayed onset (from 50 to 70% of the movement time, all *p* < 0.03, all the other *p* > 0.55) in comparison to SHAM. Again, effects in stable target trials were never observed (all *p* > 0.10, Fig. S4).

Other significant effects of ANOVA and comparisons between ED of shifted target and stable target trials in the different stimulation conditions have been reported in the Supplementary data (text and Figs S5–S7).

## Discussion

Using rTMS, here we show the first causal evidence that the medial posterior part of the human PPC—putatively the hV6A—plays a causal role in visually guided online control of reaching corrections. Indeed, we found that stimulation of hV6A during arm movement execution transiently disrupted movement adjustments to shifted locations of targets in distance and direction (vertical and horizontal axes), leaving unaffected movements toward stationary targets (i.e., the effects were specifically found when reprogramming of a movement was required). The effects consisted in time-dependent changes of the amount of deviation from the original trajectory. The impairments were dependent on the spatial position of the new target location, that is, on the type of deviation that the correction required either in the horizontal or vertical direction (direction-distance).

In both the NEAR and FAR arrangements, selective impairments of trajectory caused by the stimulation of hV6A were observed during vertical corrections (Figs. 2, 3). The impaired update of reaching in the horizontal and vertical direction observed in the present study is consistent with monkey studies showing that many cells in V6A are modulated by reaching direction and depth, with the majority of V6A cells more strongly modulated by the depth of reaching (Hadjidimitrakis et al. 2014; Bosco et al. 2016). The stronger effects of hV6A stimulation were found for corrections involving maximal arm extensions, as evident by looking at the ‘farther’, ‘leftward’ positions of FAR arrangement and at the ‘rightward’ and ‘farther’ positions of Experiment 1 (Fig. 2) and ‘farther’ positions of Experiment 2 (Fig. 3). This could be seen as a strong involvement of hV6A in integrating visual with proprioceptive input, particularly effective when a reaching correction asks for larger arm extension.

In farther conditions of both arrangements of Experiment 1, a change in Euclidean distance is evident after hV6A stimulation, though the change was different depending on the direction of the reaching corrections. It is worthwhile to note that in Experiment 1 we have tested many spatial locations, but with a small number of trials for each location, to maintain participant’s fatigue during the experimental sessions within acceptable limits. Experiment 1 produced informative, though preliminary, data which needed a confirmation with a higher number of trials for each location, so we designed the Experiment 2, with a smaller number of locations and higher number of trials for each location. As predicted, the results of Experiment 2 results were more robust and consistent, as evident in Fig. 3. In addition, in Experiment 2, the differences in sign of the deviations of farther positions was no more present. In other words, following hV6A stimulation, we consistently observed changes in performance in both experiments, providing direct evidence that hV6A is critical for correcting reaching trajectories, especially along the vertical direction.

### Time course of impairment after hV6A stimulation

Motor control theories suggest that the initial phase of reaching movement is ballistic and relies mainly on feedforward processes, because sensory signals (visual, somatosensory) used to estimate the state of the limb are not yet available, as they need at least 100–200 ms to be processed. The subsequent phase of reaching (after the peak of acceleration), on the other hand, relies on both feedforward and feedback processes (Jeannerod 1988; Prablanc and Martin 1992; Miall and Wolpert 1996; Todorov 2004). The present results show that the effect of hV6A stimulation on arm reaching trajectory was time-dependent, becoming evident only after the peak of acceleration while leaving the first, ballistic phase of the movement unaffected. In other words, the present data suggest that hV6A is involved in the intermediate part of the movement, when sensory feedbacks exert their maximum effect.

The prominent theory of optimal feedback control (Todorov and Jordan 2002; Todorov 2004) suggests that sensorimotor gains are used to adapt movement trajectories for successful reaching, and recent studies have shown that those gains are modulated following a specific time-course (Dimitriou et al. 2013; Voudouris and Fiehler 2021). In the visual domain, the gain peaks at around halfway through the movement, and then the feedback response decreases rapidly toward the end of movement, regardless of movement duration (Dimitriou et al. 2013). In the somatosensory domain, sensory processes are hampered in the early and late stages of reaching, whereas they recover around the time of peak velocity, in the intermediate stages of the movement (Voudouris and Fiehler 2021). Interestingly, here we show that stimulation of hV6A affects the reaching trajectories specifically during this intermediate phase of the movement, i.e., when visuomotor gains are maximal and somatosensory processes are supposed to recover from suppression. Conversely, the stimulation did not produce effects at the beginning, when the movement is associated with maximal somatosensory suppression and minimal visuomotor gains. Thus, the current data suggest that hV6A (similarly to monkey V6A, (Fattori et al. 2017)) is involved in the state estimation process in which sensory and motor signals interact to monitor the movement (Medendorp and Heed 2019), and is causally implicated in correcting reaching. The specificity of the effects of the stimulation of hV6A on shifted target trials is consistent with these suggestions, because a target jump challenges the estimation of the moving arm’s state, maybe because of an increased motor noise induced by a corrective motor command (Harris and Wolpert 1998). In this case, the unreliable state estimation may be compensated by enhanced somatosensory processes (Voudouris and Fiehler 2021). Thus, hV6A may have a role in this increased sensorimotor processing requested during corrective movements.

### Different role of medial and lateral sectors of PPC in reaching

In the present study, we did not see any trajectory impairments in stable target trials, whereas we observed trajectory impairments during reaching corrections in shifted target trials. This suggests that hV6A is specifically involved in the reaching reprogramming required to change the motor plan during in-flight corrections, and not merely in reaching execution.

The impairments shown in the present study are different from the effects previously observed following lateral PPC stimulation (Desmurget et al. 1999). Indeed, we did not find alterations in reaching precision nor in accuracy (see Supplementary materials), whereas Desmurget and coworkers found the opposite trend, with impairments in reaching precision accompanied by a total absence of reaching corrections (Desmurget et al. 1999) despite TMS being delivered early on in the movement. The difference between our results and those of Desmurget might be caused by several reasons. First, there are differences in the task, because in Desmurget’s task the target jump was not perceived by participants because it occurred during a saccade, whereas in our task the gaze was kept still during the target jump, and therefore the jump was perceived. Second, Desmurget used a single pulse paradigm whereas here we used rTMS. Finally, and more importantly, the PPC stimulation site was lateral in Desmurget’s study and medial in our study. The medial and the lateral parts of PPC may participate differently in reaching control. The postero-medial part could be more involved in reach planning and reprogramming, whereas the antero-lateral part might play more of a role in reaching execution and in the final adjustments of the movement. This suggestion is consistent with impairments in reach planning recently found after single pulse TMS delivered over hV6A during reaching reaction time (Breveglieri et al. 2021), as well as with recent monkey studies in which different sectors of PPC were inactivated by muscimol injections. Regarding the latter studies, it was shown that when the injection involved more antero-lateral sectors within the intraparietal sulcus (specifically area MIP), no effects in reaction times were reported, while effects of reaching accuracy and amplitude were evident and significant (Hwang et al. 2012; Christopoulos et al. 2015), suggesting a more pronounced involvement of this cortical sector in reaching execution than in planning. On the contrary, injections involving more postero-medial sites within the intraparietal sulcus (Yttri et al. 2014; Mooshagian et al. 2022), specifically at the border between area V6A and area MIP, were followed by a significant increase in reaction time and no effects on reaching accuracy, suggesting a more pronounced involvement in reach planning. The impairments we found here, only in shifted target trials when an update of the reach plan was required, strongly support this view.

### Effects of V1/V2 stimulation

We also found effects on shifted trajectories after stimulation of V1/V2, a site we used as a control in the first Experiment. Actually, a TMS coil positioned 2 cm above the inion it is likely to stimulate V1/V2 over both hemispheres (Romei et al. 2016; Chiappini et al. 2018). By doing this, we have likely impaired the bilateral representation of the lower visual field and the horizontal meridian of early visual areas such as V1-V2, but also, probably, a part of V3 (Tootell et al. 1998; Benson et al. 2014). Also, the effects of stimulation were restricted to corrections in specific horizontal directions, were time-dependent, and were earlier than the effects of hV6A stimulation. The effects were found during the rightward corrections of the NEAR targets and the rightward and leftward corrections of the FAR targets. These impairments might be caused by a visual masking effect, known to be evident if the interference given by TMS falls in a time window centered 100 ms after the visual stimulation (Amassian et al. 1989; de Graaf et al. 2014). Here, we stimulated from 50 to 250 ms after movement onset, thus within the time window of the visual masking effect induced by occipital TMS. The spatial specificity of the effects could be explained by the fact that the moving hand falls into specific parts of the retinotopic map of cortical visual areas. In particular, the effect seen during the rightward corrections of the NEAR targets could be caused by interference with visual processing of the hand moving along the contralateral horizontal meridian toward higher eccentricity values. Regarding the FAR arrangement of targets, the effects are limited to the first part of the movement, when the hand is moving across the lower visual field. We did not find effects for corrections in which the target jumped along the vertical meridian (farther and nearer corrections in the FAR arrangement), probably because the vertical meridian is not represented in the stimulated part of the occipital cortex. We thus suggest that this visual masking effect could interfere with the online guidance of movements, preventing a correct flow of visual information from the early visual areas to the areas of the dorsal stream. Further experiments with a higher number of shifted target trials and specifically aimed at investigating the functions of early visual areas in reaching, or in a specific visual recognition task, are needed to clarify this issue and to better interpret these data. Nevertheless, our findings provide preliminary evidence of a double dissociation between occipital and hV6A stimulation and show a specific timing of causal involvement of these brain regions in online reaching corrections, ruling out the influence of unspecific effects of the stimulation.

## Conclusions

This study highlights the critical role of a postero-medial sector of the human PPC—putatively hV6A—in action reprogramming during a reaching task. We found hV6A to be relevant to online reaching adjustments in both direction and distance from the body. Since these adjustments require enhanced state estimation processes, the current results suggest that hV6A is critically involved in these activities.

### Supplementary Information

Below is the link to the electronic supplementary material.

Supplementary file1 (DOCX 37233 KB)

## Data Availability

The datasets generated and/or analysed during the current study are not publicly available since the informed consent signed by the volunteers enrolled in the study did not contain the possibility to share the data publicly. Nevertheless, data are available from the corresponding author on reasonable request.
