# Supplementary material for: rTMS over the human medial parietal cortex impairs online reaching corrections
Source: Brain Struct Funct. 2023 Dec 23;229(2):297–310. doi: 10.1007/s00429-023-02735-7 (PMC10917872; doi:10.1007/s00429-023-02735-7)
Supplement: Supplementary file 1 — Supplementary file1 (DOCX 37233 KB) [file 429_2023_2735_MOESM1_ESM.docx]

**Supplementary data**

**rTMS over the human medial parietal cortex in humans impairs online reaching corrections.**

**Rossella Breveglieri*, Sara Borgomaneri, Annalisa Bosco, Matteo Filippini, Marina De Vitis, Alessia Tessari, Alessio Avenanti, Claudio Galletti, Patrizia Fattori.**

*** Corresponding author: rossella.breveglieri@unibo.it**

**
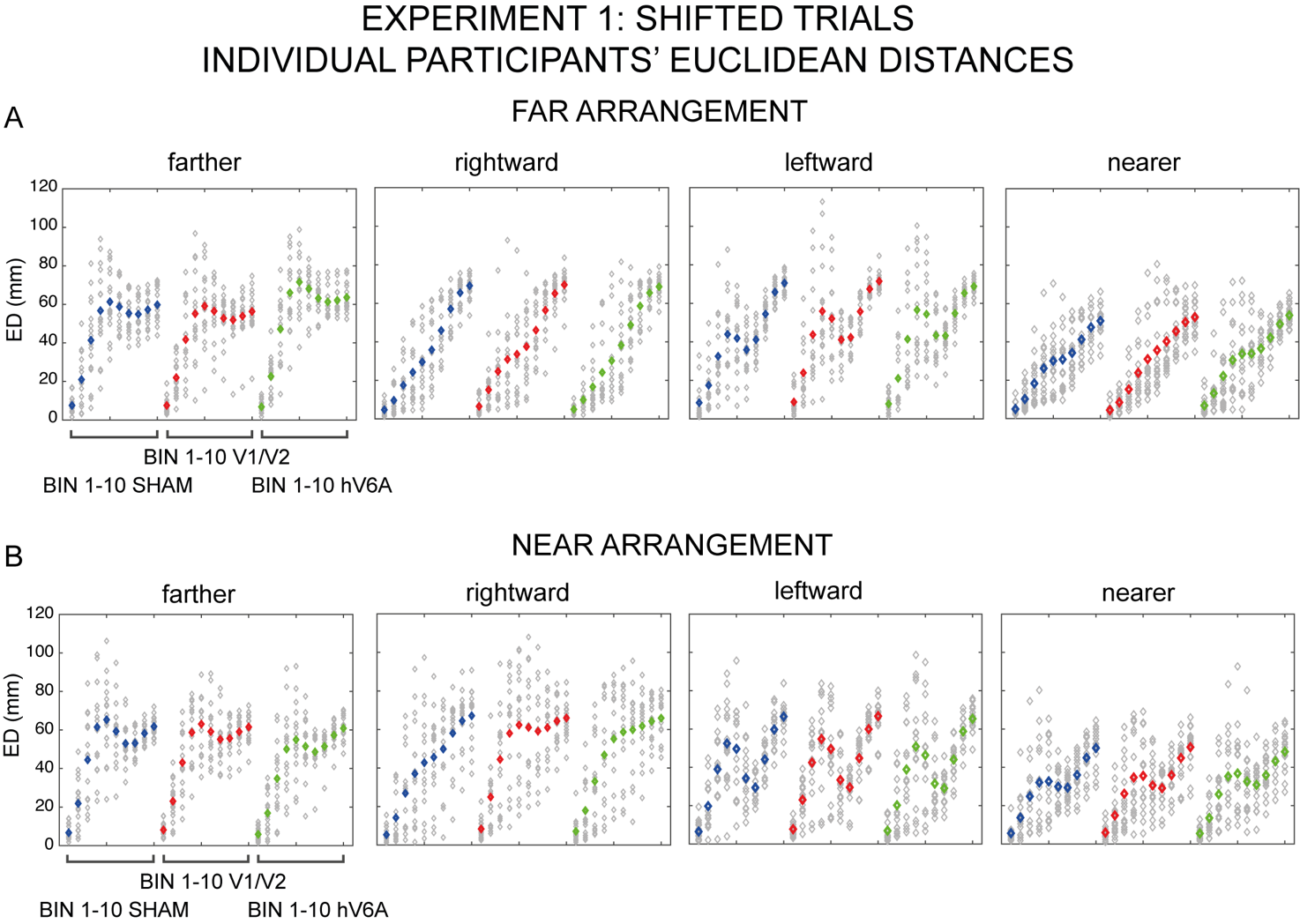
**

Figure S1

*Experiment 1: individual participants’ ED in shifted target trials*

Euclidean distance (ED) between the deviated trajectory in shifted target trials and the corresponding central one for each individual participant (gray diamonds) and group mean values (blue diamonds, Sham, red diamonds, V1/V2, green diamonds, hV6A) for FAR arrangement of targets (A) and for NEAR arrangement of targets (B). Within each arrangement, data are sorted according to ED extent and are divided between horizontal/vertical direction of correction, stimulation sites (Sham, V1/V2, hV6A) and time bins. Note that the calculation of the ED has been performed using the 3D coordinates of each trajectory point. Other conventions as in Figs. 2-3.


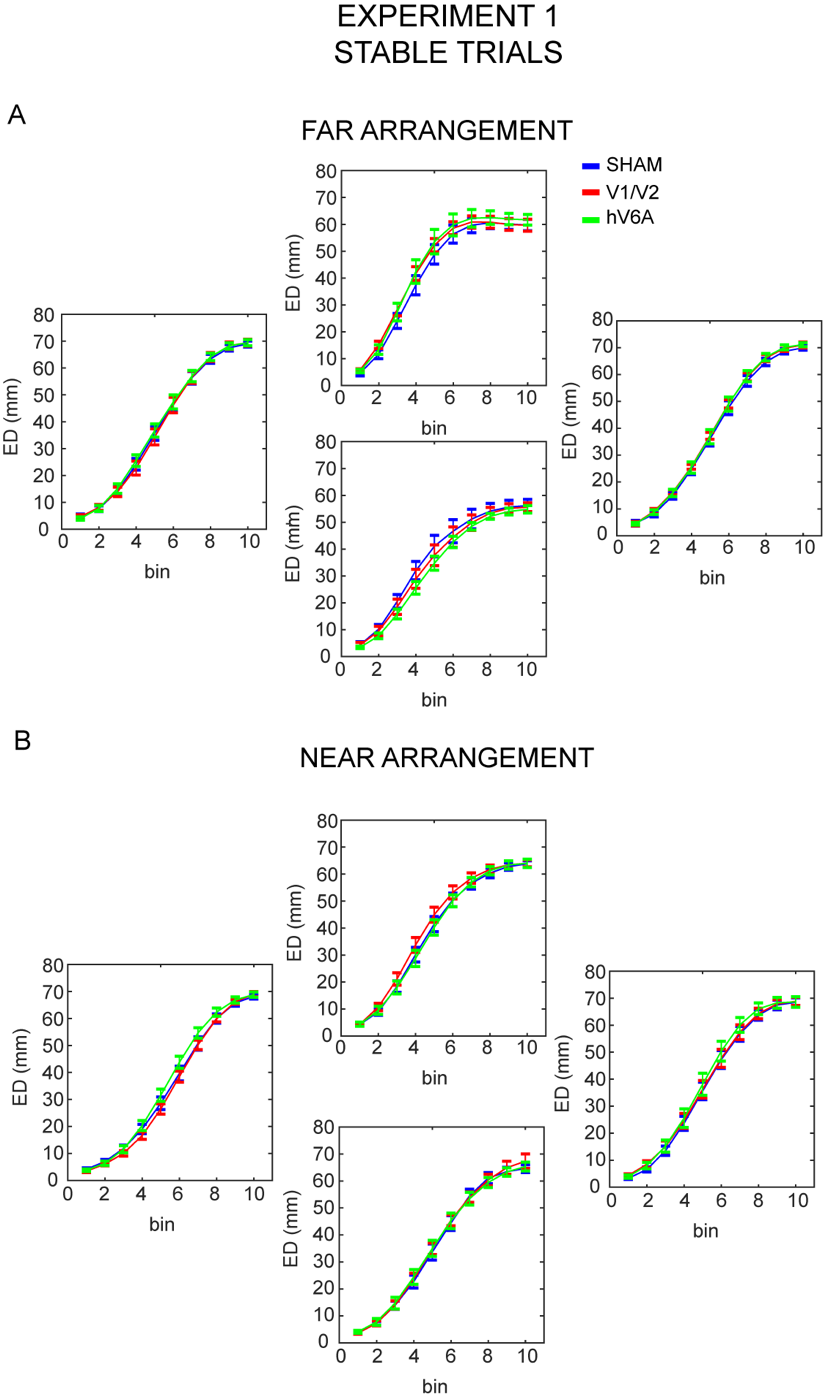


Figure S2

*Experiment 1 stable target trials: ED values.*

Mean population ED between the trajectories of stable target trials and the corresponding central one for FAR arrangement of targets (A) and for NEAR arrangement of targets (B). Within each arrangement, data are grouped as to horizontal/vertical direction of movement, stimulation sites, and time bins. No effects of TMS were found (all p>0.05). Other conventions as in Figs. 2-3.


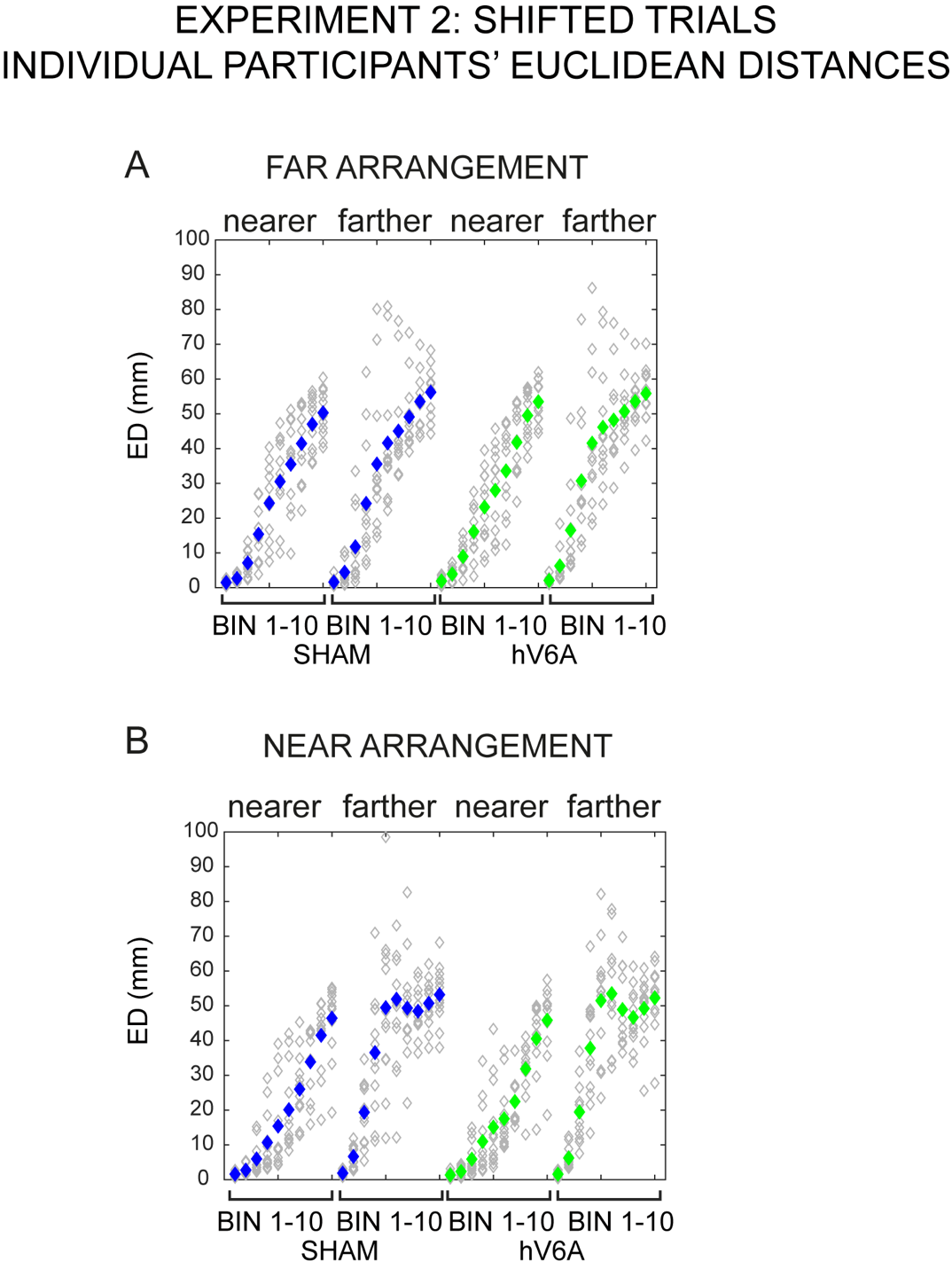


Figure S3

*Experiment 2 shifted target trials: ED values of individual participants*

Euclidean distance (ED) between the deviated trajectory in shifted target trials and the corresponding central one for each individual participant (gray diamonds) and group mean values (blue diamonds, Sham, green diamonds, hV6A) for FAR arrangement of targets (A) and for NEAR arrangement of targets (B). Other conventions as in Figs. 2-3, S1.


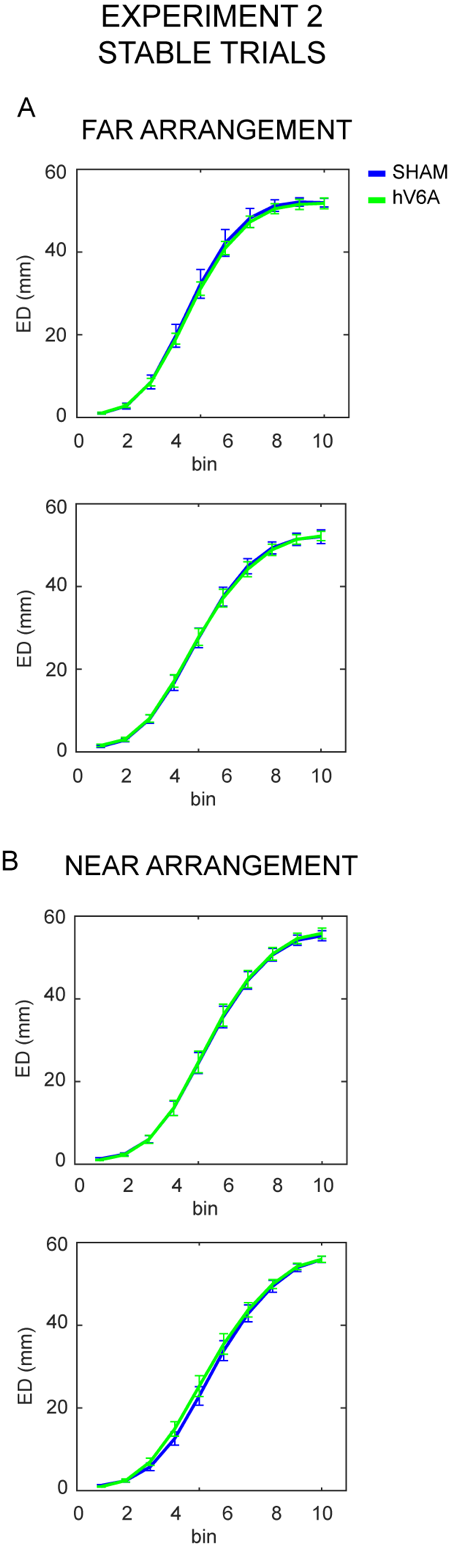


Figure S4

*Experiment 2 stable target trials: ED values.*

Mean population ED between the trajectories in stable target trials and the corresponding central one of FAR arrangement of targets (A) and of NEAR arrangement of targets (B). Other conventions as in Figs. 2-3, S2.

*Euclidean distance analysis: other significant effects*

Experiment 1: FAR targets

In addition to the 4-way interaction described in the main text, ED was influenced also by the main effect of Position (F_3,45_=25.41, p<0.001, partial η^2^=0.63), effect driven by the higher ED for farther movements compared to all the other positions (all p<0.01), by the ED of leftward movements higher than that of nearer movements (p<0.001), whereas the ED of leftward and rightward movements were not statistically different (p=0.05).

Furthermore, ED was also affected by the Time bin (F_9,135_=508,07, p<0.001, partial η^2^=0.97), with an increasing trend of ED from the first to the last bin (all p<0.01 except bin 9 vs bin 10, p=0.11).

In addition, some interactions were significant:

1) the interaction Type of trial by Position (F_3,45_=11.73, p<0.001, partial η^2^=0.44) with the increasing ED from nearer to rightward to leftward to farther corrections in shifted target trials (all p<0.01). The same trend was observed in stable target trials (all p<0.04 except the comparison leftward versus rightward and nearer which were not significant (all p>0.08)).

2) the interaction Type of trial by Time bin (F_9,135_=36.73, p<0.001, partial η^2^=0.71), effect driven by the higher ED of shifted target trials in bins 2 to 5 (all p<0.03) and higher ED of stable target trials in bin 6 to 9 (all p<0.03, bins 1 and 10 p>0.11).

3) the interaction Position by Time bin (F_27,405_=28.57, p<0.001, partial η^2^=0.66), driven by the higher ED during farther or leftward movements compared to nearer ones from bin 2 to 10 (all p<0.01), whereas ED for rightward movement was higher than nearer ones from bin 6 to 10 (all p<0.04).

4) the interaction Type of trial by Position by Time bin (F_27,405_=11.31, p<0.001, partial η^2^=0.43). In this effect, the ED of leftward movements was higher during shifted target trials than stable ones from bin 2 to 5 and lower during bins 6-8 (all p<0.01, all the other bins p>0.05). A similar trend was observed during farther trials but only in the first half of the movement (ED of perturbed trials higher than unperturbed in bins 2-5 all p<0.01, the remaining bins p>0.08). During nearer movements, the ED of shifted target trials was lower than of stable ones in bins 5-9 (all p<0.01). The same trend, significant in bins 5-8 (all p<0.01), was observed in rightward movements.

Experiment 1: NEAR targets

ED was influenced also by the main effect of Position (F_3,45_=16.00, p<0.001, partial η^2^=0.52), effect driven by the lower ED for nearer movements (all p<0.02); ED of leftward movements was lower than rightwards and farther ones (all p<0.01) and higher than nearer ones (p=0.02). ED of rightward movements was not significantly different from ED of farther movements (p=0.69) and was significantly different from all the other movements (all p<0.01).

We found also the significant main effect of Time bin (F_9,135_=308.77, p<0.001, partial η^2^=0.95) and an increasing trend of ED from the first to the last bin (all p<0.001 except the comparison between bin 5 and 6, p=0.10 and bin9 vs bin 10, p=0.07).

Several interaction effects were significant.

1) the interaction Type of trial by position (F_3,45_=10.12, p<0.001, partial η^2^=0.40), effect driven by the increasing trend of ED in nearer-leftward-rightward-farther movements (all p<0.02 except the comparison between rightward and farther, p=0.82) only in shifted target trials (in stable target trials the comparisons between ED of the different movements were all non-significant, all p>0.15).

2) the interaction Type of trial by Time bin (F_9,135_=54.48, p<0.001, partial η^2^=0.78), supported by higher ED values in shifted target trials in bin 2-5 (all p<0.001) and the opposite trend in bins 7-10 (all p<0.001).

3) the interaction Position by Time bin (F_27,405_=15.94, p<0.001, partial η^2^=0.52), driven by the higher ED values for rightward and farther movements compared to nearer and leftward movements in bins 5-9 (all p<0.01).

4) the interaction Stimulation condition by Position by Time bin (F_54,810_=1.79, p<0.001, partial η^2^=0.11), driven by higher ED after V1/V2 or hV6A stimulation than Sham during rightward movements in bins 2-6 (V1/V2, all p<0.05) or bins 3-7 (hV6A, all p<0.05), that in turn were different only in bin 3 (p=0.009). During farther movements, lower values of ED after hV6A stimulation have been observed (bins 3-5 all p<0.01 compared to SHAM and to V1/V2 stimulation).

5) the interaction Trial type by Position by Time bin (F_27,405_=9.15, p<0.001, partial η^2^=0.38), supported by higher ED values during shifted target trials during the first part of nearer movements (bin 2-4, all p<0.01), whereas the opposite trend was seen in the subsequent movement parts (bin 6-10, all p<0.01). During rightward movements, the ED was higher in shifted target trials (bins 2-6, all p<0.01). During leftward movements, the ED was higher in shifted target trials (bins 2-5 all p<0.01) whereas the opposite was true in subsequent bins (bins 6-9, all p<0.01). Lastly, during farther movements, ED was higher in shifted target movements in bins 2-5 (all p<0.01) and lower in bin 8 (p<0.001).

Experiment 2: FAR targets

We have found a significant main effect of Time bin (F_9,99_=623.21, p<0.001, partial η^2^=0.98), effect driven by the increasing ED across subsequent time bins (all p<0.01 except the comparisons between bins 1-2, p=0.06, and bins 9-10, p=0.20).

In agreement with the results of Experiment 1, significant interactions have been found.

1) the interaction Type of trial by Time bin (F_9,99_=6.30, p<0.001, partial η^2^=0.36), driven by higher ED values in shifted target trials (bin 3-4, all p<0.01) and the opposite in bins 6-8 (all p<0.01). 2) the interaction Position by Time bin (F_9,99_=3.59, p<0.001, partial η^2^=0.25), supported by higher values of ED for farther movements than for nearer ones (bins 4-8, all p<0.05).

Experiment 2: NEAR targets

We found the significant main effect of Position (F_1,11_=23.85, p<0.001, partial η^2^=0.68), supported by the higher ED values for farther movements. In addition, the main effect of Time bin was significant (F_9,99_=331.06, p<0.001, partial η^2^=0.97), driven by the increasing ED across subsequent time bins (all p<0.01 except the comparisons between bins 1-2, p=0.19, and bin 9-10, p=0.08).

Several interactions were significant.

1) the interaction Trial type by Position (F_1,11_=71.41, p<0.001, partial η^2^=0.87) driven by higher ED values for shifted target movements toward farther targets (p<0.001). In stable target trials, no difference between positions have been found (p=0.76).

2) the interaction Stimulation condition by Time bin (F_9,99_=3.29, p<0.01, partial η^2^=0.23), driven by higher values of ED after hV6A stimulation in intermediate phases of the movement (bin 4-6, all p<0.02).

3) the interaction Trial type by Time bin (F_9,99_=14.05, p<0.001, partial η^2^=0.56), driven by higher ED values in shifted target trials (bin 3-5, all p<0.01) and the opposite trend in the last parts of the movement (bin 7-10, all p<0.01).

4) the interaction Position by Time bin (F_9,99_=19.75, p<0.001, partial η^2^=0.64) supported by higher ED values for farther movements in the central phases (bin 3-9, all p<0.02).

5) the interaction Trial type by Position by Time bin (F_9,99_=41.35, p<0.001, partial η^2^=0.79), driven by higher ED values in stable target nearer movements (bin 4-10, all p<0.03), whereas the opposite was true for farther movements (bins 2-7, all p<0.04).

The following figures S5-7 represent the comparisons between ED of the shifted target and stable target trials in all the stimulation conditions.


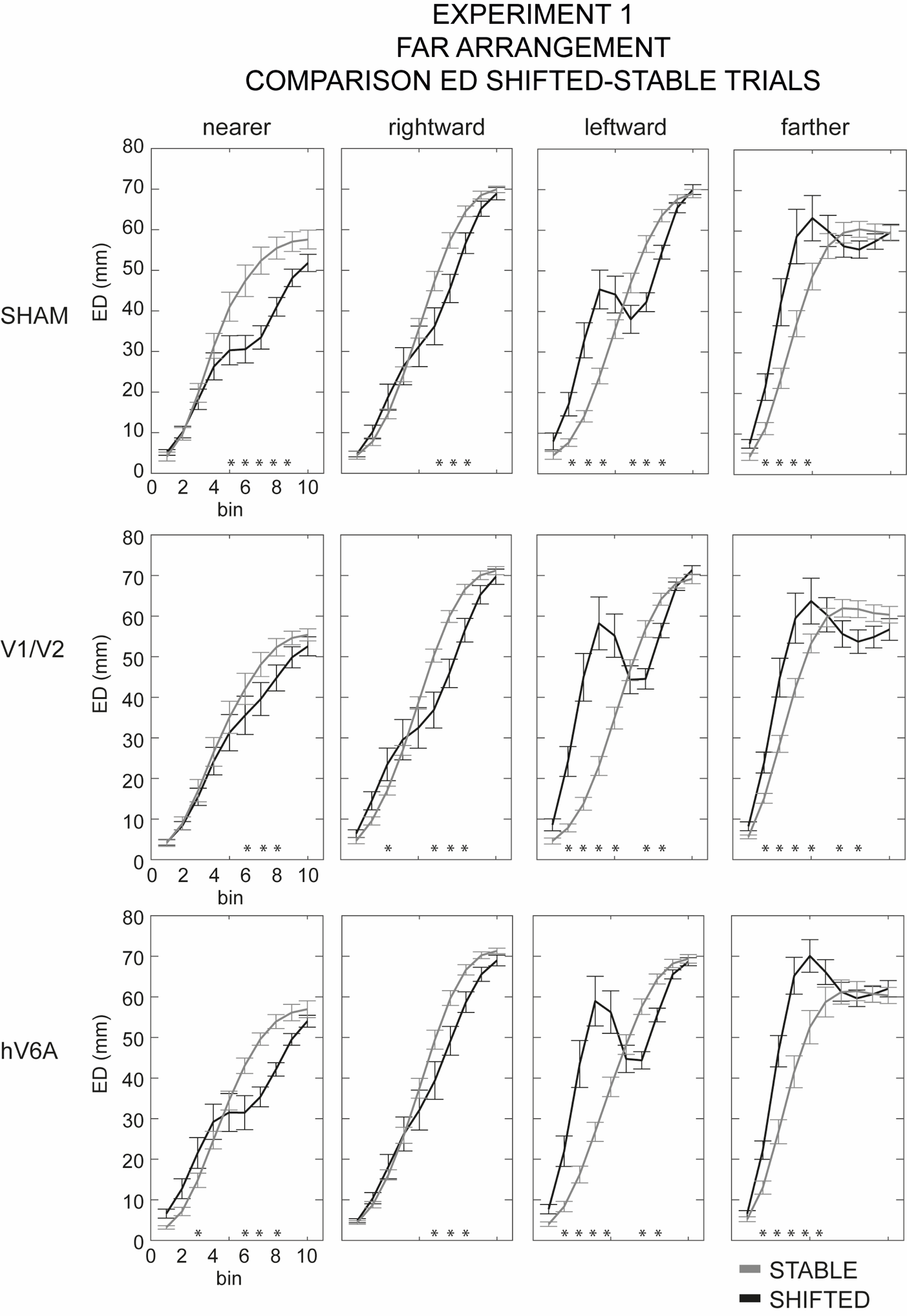


Figure S5

Comparison between ED of shifted target and stable target trials in the different stimulation conditions of Experiment 1, FAR arrangement. Asterisks represent significant posthoc comparisons (p<0.05). Conventions as in Figs. 2, 3. Stimulations of V1/V2 and of hV6A were effective in modulating the differences between ED of shifted target and stable target trials, but the general trend was maintained.


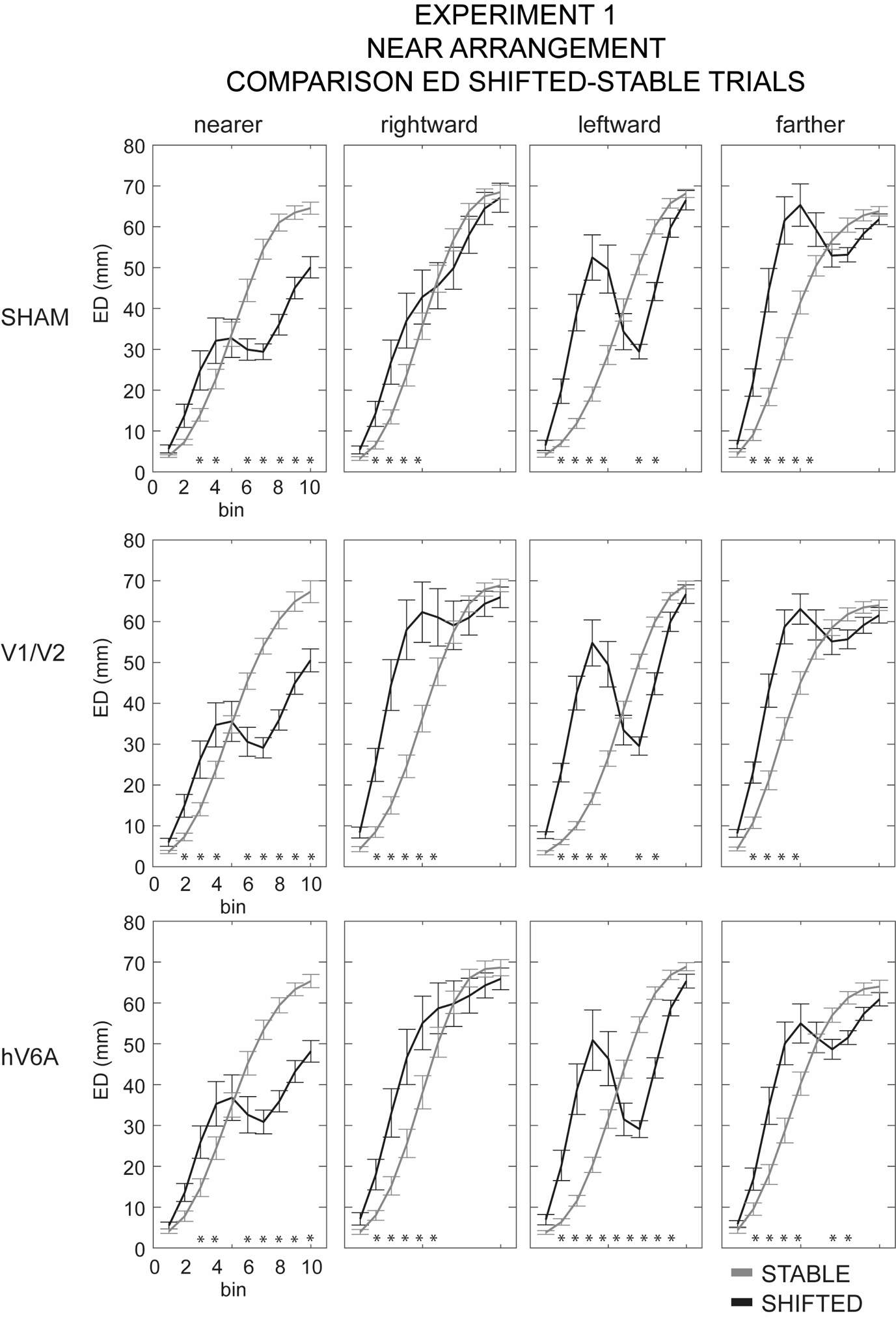


Figure S6

Comparison between ED of shifted target and stable target trials in the different stimulation conditions of Experiment 1, NEAR arrangement. Conventions as in Figs. 2, 3, S5. Stimulations of V1/V2 and of hV6A were effective in modulating the differences between ED of shifted target and stable target trials, but the general trend was maintained.


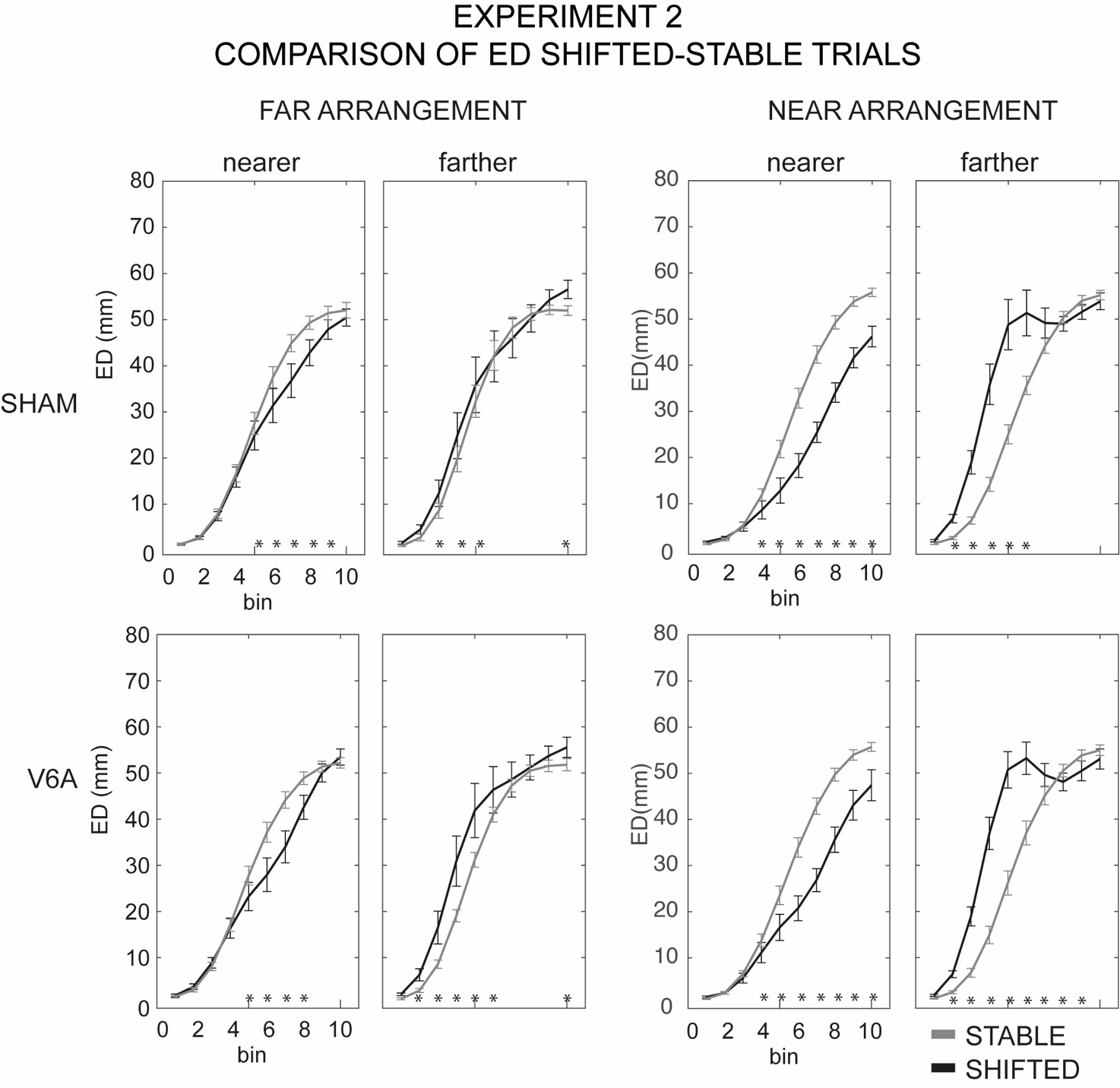


Figure S7

Comparison between ED of shifted target and stable target trials in the different stimulation conditions of Experiment 2 (both arrangements). Conventions as in Figs. 2, 3, S5, S6. Stimulations of V1/V2 and of hV6A were effective in modulating the differences between ED of shifted target and stable target trials, but the general trend was maintained.

*Effects on reaching precision and accuracy.*

We were also interested in investigating the effect of rTMS on the encoding of the reaching goal, considering the result of the reaching movement at its end: movement precision and accuracy. Movement accuracy and precision were extracted from the endpoints recorded by the touchscreen and derived from the parameters of 95% confidence ellipses fit to hand position (endpoint) distributions measured at movement offset, as performed in previous studies (Vesia et al. 2006, 2010; Prime et al. 2008; Breveglieri et al. 2021). Constant error (accuracy) was calculated by taking the signed difference between the horizontal (horizontal error) and vertical (vertical error) coordinates of the center of movement ellipses and of each target location. Variable error (precision) was measured using the area of these ellipses (Vesia et al. 2006, 2010; Prime et al. 2008; Breveglieri et al. 2021). Statistical reliability of differences between mean constant errors, variable errors, and movement times were tested for each arrangement of targets (FAR and NEAR), by performing a three-way repeated measures ANOVA with the following factors: Stimulation site (3 levels: Sham, V1/V2, hV6A in Experiment 1, 2 levels: Sham, hV6A in Experiment 2), Trial Type (2 levels: stable target, shifted target), Type of Perturbation (4 levels, positions of targets in each arrangement in Experiment 1, 2 levels, positions of targets in each arrangement in Experiment 2, Fig. 1A).

In keeping with the absence of effects of TMS in the last phase of arm movement shown in the main manuscript, the stimulation of hV6A did not affect the accuracy or the precision of reaching, as explained hereafter.

*Reaching precision: variable error*

*Experiment 1*

Reaching precision was not affected by TMS in the FAR arrangement (all F < 1.77, all p > 0.19, all partial η^2^<0.10) or in the NEAR arrangement (all F < 1.21, all p > 0.31, all partial η^2^ <0.9).

*Experiment 2*

Reaching precision was not affected by TMS in the FAR arrangement (all F < 1.15, all p >0.45, all partial η^2^ <0.02), nor was it affected in the NEAR arrangement (all F <0.86, all p >0.37, all partial η^2^ <0.01).

*Reaching accuracy: constant (horizontal) error*

*Experiment 1*

The analysis of horizontal errors in the FAR positions revealed a significant Stimulation site by Type of Perturbation interaction (F_(6,90)_= 2.36, p = 0.04, partial η^2^=0.14). However, the posthoc comparisons did not reveal any significant differences in the direction errors in any of the positions according to the stimulation (Sham vs. V1/V2 all positions p>0.11, Sham vs. hV6A all positions p>0.61, V1/V2 vs. hV6A all positions p>0.20). In the NEAR positions, the horizontal errors were not influenced by the stimulation (all F<2.18, all p>0.12).

*Experiment 2*

Horizontal errors were not affected by TMS in the FAR arrangement (all F < 0.33, all p >0.31, all partial η^2^ <0.01) nor in the NEAR arrangement (all F <3.47, all p >0.09, all partial η^2^<0.06).

*Reaching accuracy: constant (vertical) error*

*Experiment 1*

Vertical errors were not influenced by the stimulation, either in the FAR positions (all F < 2.09, all p > 0.06, all partial η^2^ <0.12) or in the NEAR ones (all F < 1.11, all p > 0.34, all partial η^2^ <0.07).

*Experiment 2*

Vertical errors were not influenced by the stimulation, either in the FAR positions (all F < 2.94, all p > 0.11, all partial η^2^ <0.04) or in the NEAR ones (all F < 2.39, all p > 0.07, all partial η^2^ <0.03).

**Supplementary references**

Breveglieri R, Bosco A, Borgomaneri S, et al (2021) Transcranial Magnetic Stimulation Over the Human Medial Posterior Parietal Cortex Disrupts Depth Encoding During Reach Planning. Cereb Cortex 31:267–280. https://doi.org/10.1093/cercor/bhaa224

Prime SL, Vesia M, Crawford JD (2008) Transcranial magnetic stimulation over posterior parietal cortex disrupts transsaccadic memory of multiple objects. J Neurosci 28:6938–6949. https://doi.org/10.1523/JNEUROSCI.0542-08.2008

Vesia M, Monteon JA, Sergio LE, Crawford JD (2006) Hemispheric asymmetry in memory-guided pointing during single-pulse transcranial magnetic stimulation of human parietal cortex. J Neurophysiol 96:3016–27. https://doi.org/10.1152/jn.00411.2006

Vesia M, Prime SL, Yan X, et al (2010) Specificity of human parietal saccade and reach regions during transcranial magnetic stimulation. J Neurosci 30:13053–65. https://doi.org/10.1523/JNEUROSCI.1644-10.2010
